# *QuickStats:* Percentage of Total Deaths, by Age and Hispanic Origin and Race*^,^^†^ — United States, 2020

**DOI:** 10.15585/mmwr.mm7137a6

**Published:** 2022-09-16

**Figure:**
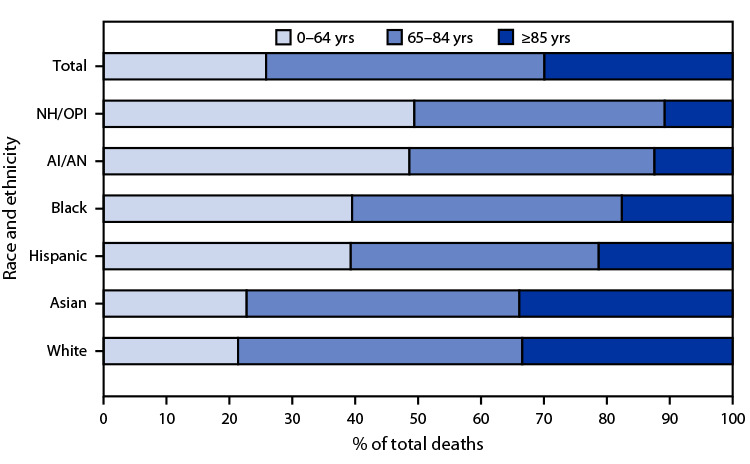
Significant differences in the age distribution of deaths by race and ethnicity were observed in the United States during 2020. Decedents aged <65 years accounted for 26% of all U.S. deaths, but they accounted for approximately 50% of deaths among AI/AN and NH/OPI persons, 40% of deaths among Black or African American (Black) and Hispanic or Latino (Hispanic) persons, and 20% of deaths among Asian and White persons. Smaller differences were noted among persons aged 65–84 years. Among persons aged ≥85 years, the pattern was reversed, with the percentage of all deaths ranging from approximately 11% among AI/AN and NH/OPI persons to 33% for Asian and White persons.

**Source:** National Vital Statistics System, Underlying Cause of Death by Single-Race Categories, 2018–2020. https://wonder.cdc.gov/ucd-icd10-expanded.html

